# The protein phosphatase 2A catalytic subunit StPP2Ac2b enhances susceptibility to *Phytophthora infestans* and senescence in potato

**DOI:** 10.1371/journal.pone.0275844

**Published:** 2022-10-10

**Authors:** María N. Muñiz García, Cecilia Grossi, Rita M. Ulloa, Daniela A. Capiati

**Affiliations:** Instituto de Investigaciones en Ingeniería Genética y Biología Molecular “Dr. Héctor Torres”, Consejo Nacional de Investigaciones Científicas y Técnicas (CONICET), Buenos Aires, Argentina; Institute for Sustainable Plant Protection, C.N.R., ITALY

## Abstract

The serine/threonine protein phosphatases type 2A (PP2A) are involved in several physiological responses in plants, playing important roles in developmental programs, stress responses and hormone signaling. Six PP2A catalytic subunits (StPP2Ac) were identified in cultivated potato. Transgenic potato plants constitutively overexpressing the catalytic subunit *StPP2Ac2b* (StPP2Ac2b-OE) were developed to determine its physiological roles. The response of StPP2Ac2b-OE plants to the oomycete *Phytophthora infestans*, the causal agent of late blight, was evaluated. We found that overexpression of *StPP2Ac2b* enhances susceptibility to the pathogen. Further bioinformatics, biochemical, and molecular analyses revealed that StPP2Ac2b positively regulates developmental and pathogen-induced senescence, and that *P*. *infestans* infection promotes senescence, most likely through induction of *StPP2Ac2b* expression.

## Introduction

The serine/threonine protein phosphatases type 2A (PP2A) are implicated in several physiological processes in plants [1], including response to different stresses [2–6], developmental programs [7, 8], and hormone signaling [9–13]. PP2A comprises a catalytic (C), a scaffolding (A) and a regulatory (B) subunit. The holoenzyme exists as a dimer of C and A subunits, or as a trimer consisting of C, A and B subunits. The *Arabidopsis thaliana* genome encodes five catalytic subunits, which are clustered into two subfamilies (I and II), three scaffolding subunits and 17 regulatory subunits [14, 15].

An increasing number of reports indicate that PP2A plays a central role in plant resistance and immunity, mainly in response to fungal pathogens. These studies are based on silencing, knockout or overexpression of PP2A catalytic, scaffolding or regulatory subunits in different plant species. Silencing of the subfamily I of PP2A catalytic subunits in *Nicotiana benthamiana* increases the hypersensitive response to effector proteins from the biotrophic fungus *Cladosporium fulvum* and the bacterium *Pseudomonas syringae* [16]. In the same way, Zhu et al. [17] demonstrated that silencing of a wheat PP2A catalytic subunit (TaPP2Ac-4D) enhances resistance to the necrotrophic fungus *Rhizoctonia cerealis*. In contrast, Lin et al. [18] showed that in rice, knockout of *OsPP2A-1*, which encodes a catalytic subunit, increased susceptibility to the necrotrophic pathogen *Rhizoctonia solani*, while its overexpression confers resistance. Regarding scaffolding subunits, Chen et al. [19] reported that silencing of *PP2Aa* in *N*. *benthamiana* decreases resistance to the hemibiotrophic oomycete *Phytophthora capsici*.

The regulatory subunit PP2A-B´γ has been implicated in the control of defense responses, as evidenced by the phenotypic analysis of the *pp2a-b´γ* mutant, which shows an increased resistance to the necrotrophic fungal pathogen *Botrytis cinerea* [20]. Recently, Durian et al. [21] reported that PP2A-B´γ regulates *B*. *cinerea* resistance by negatively regulating the expression of salicylic acid-related defense genes. Interestingly, PP2A-B´γ also delays initiation and progression of leaf senescence at late stages of development [21], however the relationship between senescence and pathogen resistance/susceptibility is still unclear.

In cultivated potato (*Solanum tuberosum*), six PP2A catalytic isoforms were identified and named StPP2Ac1, 2a, 2b, 3, 4 and 5 [22]. The expression of the subfamily I members (*StPP2Ac1*, *2a* and *2b*) is regulated in response to environmental conditions, while members of the subfamily II (*StPP2Ac3*, *4* and *5*) show more invariant and constitutive expression patterns, suggesting housekeeping functions for the encoded proteins [22, 23]. The data from expression analyses, together with studies based on the use of protein phosphatase inhibitors, suggest that PP2A catalytic isoforms of the subfamily I could be involved in the response to fungal elicitors, abiotic stress and tuber development [22, 23]. Transgenic potato plants constitutively overexpressing *StPP2Ac2b* (StPP2Ac2b-OE) were developed to determine the physiological roles of this isoform. In a previous study, analyzing the phenotype of StPP2Ac2b-OE lines, we showed that StPP2Ac2b acts in stolons as a positive regulator of tuber induction, integrating different tuberization-related signals mainly through the modulation of gibberellic acid/abscisic acid ratio [24]. In the present work, we evaluated the response of StPP2Ac2b-OE plants to the hemibiotrophic oomycete *Phytophthora infestans*, the causal agent of late blight, which is the most important disease of potato. We found that overexpression of *StPP2Ac2b* increases the susceptibility to *P*. *infestans*, and performed further bioinformatics, biochemical, and molecular analyses to determine the mechanism underlying susceptibility in this particular host-pathogen interaction.

## Materials and methods

### Plant material and growth conditions

Transgenic potato plants (*Solanum tuberosum* cv. Spunta) expressing the *StPP2Ac2b* gene under the control of the cauliflower mosaic virus 35S promoter (StPP2Ac2b-OE) were generated by Agrobacterium-mediated transformation [24]. Eight transgenic lines were obtained. Three representative lines (L1, L4 and L7) were selected for phenotypic analysis. L4 is morphologically similar to the wild type, while the morphological differences between L1 and L7 and the wild type are more marked [24]. L1 and L4 contain three copies of the transgene, while L7 is a single-copy transformant. L1, L4 and L7 derived from independent integration events [24]. Regenerated plants carrying no plasmid but obtained by the same regeneration method were used as controls (wild type) for phenotypic analysis. In vitro plants were obtained by micropropagation of single-node cuttings in Murashige and Skoog medium (MS medium; Prod no. M519, PhytoTechnology Laboratories, Shawnee Mission, KS, USA) containing 20 g/L sucrose solidified with 0.7% (w/v) agar, under a 16 h light photoperiod (4000 lux light intensity) at 22°C. Soil-grown plants were obtained from in vitro-propagated plants transferred to soil (ex vitro), and cultivated in a growth chamber at 20°C, under a 16 h light photoperiod (4000 lux light intensity), in 1 L pots with commercial soil mixture (Grow Mix Multipro, Terrafertil Argentina). Ex vitro plants were used for all the experiments, except for those involving infection of detached leaves (see below).

### Infection with *P*. *infestans*

Wild type and StPP2Ac2b-OE plants were inoculated with *P*. *infestans* strain 88069td [25]. Infection experiments were carried out with detached leaves and whole plants. For detached-leaf experiments, plants obtained from seed tubers cultivated in greenhouse were used. Plants were grown in 1 L pots with commercial soil mixture (Grow Mix Multipro, Terrafertil Argentina) at 22–24°C, under a 16 h light photoperiod. Four weeks after planting, the second fully expanded leaves were collected and placed in containers with tap water in sealed transparent plastic boxes to ensure high humidity, in a growth chamber (20°C, 16 h light photoperiod, 4000 lux light intensity). To rule out the effect of leaf size, leaves of similar size were selected from wild type and transgenic lines to perform this experiment. Forty eight hours after excision, leaves were infected with *P*. *infestans*; 10-μL droplets of sporangia suspension (2.5x10^4^ spores/mL) were pipetted on the abaxial side of the leaves (two equidistant spots per leaf). No visible signs of senescence were observed at the moment of inoculation. Four days post-inoculation (dpi), leaves were photographed and the necrotic lesion areas were measured using Fiji software (https://fiji.sc/); the disease severity was estimated in a scale from 1 to 10, as described in Fall et al. [26]. Three independent experiments were performed with 14 leaves per line for each condition (non-infected or infected).

For whole-plant experiments, plants transferred to soil ex vitro and cultivated in growth chamber (20°C, 16 h light photoperiod, 4000 lux light intensity) for four weeks were in infected with *P*. *infestans*; 10-μL droplets of zoospore suspension (2.5x10^4^ spores/mL) were pipetted on the abaxial side of the second and third leaves (two equidistant spots per leaf). Non-infected plants were used as controls. Plants were placed in a sealed transparent acrylic box to ensure high humidity. At four dpi, the percentage of inoculated leaves presenting extensive necrosis (diameter ≥ 5 mm) was determined. Three independent experiments were performed with 12 plants per line for each condition (non-infected or infected). StPP2Ac2b-OE L1 and L7 plants were smaller than the wild type, but there were no visible differences in plant size between L4 and untransformed plants (S1 Fig).

Non-infected leaves (from non-infected plants), and infected and distal (upper) leaves from inoculated plants were collected and frozen in liquid nitrogen for RT-qPCR analysis and determination of chlorophyll a and b. Three independent experiments were performed for each determination; a pool of three leaves from different plants were sampled for each biological replicate.

### RT-qPCR analysis

RNA isolation, cDNA synthesis and RT-qPCR reactions were carried out as described in Muñiz García et al. [24]. *EF1-α* was used as reference gene [27]. *StPP2Ac2b*, *StPP2Ac1*, *StPP2Ac2a*, *Pathogenesis-related protein 1b* (*StPR-1b*), *phenylalanine ammonia-lyase 1* (*StPAL1*), *StNAC030*, *StMYB229*, *StSGR1/NYE1*, *StPP2C31*, *StEIN3/EIL2*, *StWRKY61* and *StLOX3* mRNA levels were determined using the primers showed in S1 Table; the amount of cDNA used in each reaction was derived from 1 ng of total RNA, and reactions were carried out under the following conditions: 95°C/15 min (1 cycle); 95°C/15 s; 60°C/1 min; 72°C/30 s (40 cycles). Relative expression was calculated by the method of Pfaffl [28]. The experiments were performed three times independently, with three technical replicates for each cDNA.

### Chlorophyll content

Chlorophyll a and chlorophyll b contents were determined as described in Muñiz García et al. [29]. Alternatively, in order to preserve plant integrity, chlorophyll content was measured on fully expanded leaves using a SPAD chlorophyll meter (Clorofilio^®^, Cavadevices, Argentina).

### Statistical analysis

Statistical analysis was performed using one-way or two-way ANOVA followed by Bonferroni multiple comparisons test with GraphPad Prism version 8.4.3. Different letters in the bar graphs indicate statistically significant differences. *P* < 0.05 was considered significant.

## Results

### Expression of PP2A catalytic subunits of the subfamily I in response to *P*. *infestans* infection

**Fig 1:**
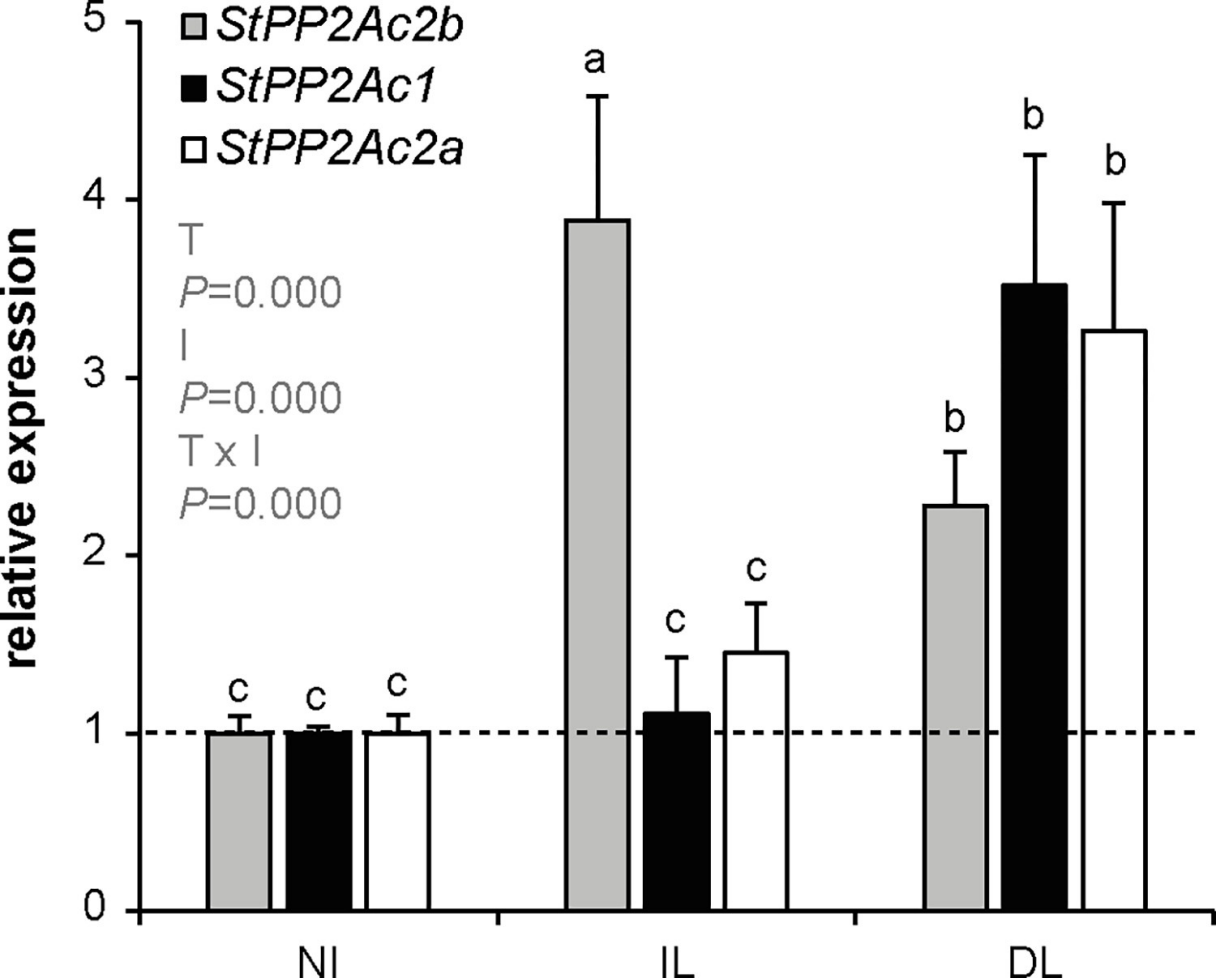
Expression analysis of *StPP2Ac2b*, *StPP2Ac2a* and *StPP2Ac1* in wild type potato plants infected with *P*. *infestans*. Plants were inoculated with *P*. *infestans* as described in Materials and Methods. After four dpi, expression was determined by RT-qPCR in leaves from non-infected plants (NI), infected leaves (IL) and distal leaves (DL), relative to NI (dashed line). Data are the mean ± SEM of of three independent experiments. Data were analyzed by two-way ANOVA, followed by Bonferroni post-hoc test (α = 0.05) comparing the expression of each gene between NI, IL and DL, and the expression of the different genes within each experimental condition (NI, IL or DL). *P*-values of the effects of treatment (T: NI, IL, DL), StPP2Ac isoform type (I: *StPP2Ac2b*, *StPP2Ac1*, *StPP2Ac2a*) and their interaction are shown. The two-way ANOVA revealed a significant interaction between the effects of T and I, and a significant effect of T and I on the expression levels of StPP2Ac.

To determine the involvement of the PP2A in the response to *P*. *infestans* infection, wild type plants were inoculated with the pathogen and the expression of *StPP2Ac2b*, *StPP2Ac1* and *StPP2Ac2a* was determined by RT-qPCR. These genes encode the three isoforms of the PP2A subfamily I catalytic subunits in potato. In infected leaves, the mRNA levels of *StPP2Ac2b* significantly increased with respect to non-infected leaves, while the expression of *StPP2Ac1* and *StPP2Ac2a* remained unchanged (Fig 1). In distal leaves, the expression of all three isoforms were induced. Although preliminary, these results suggest that PP2A catalytic subunits of the subfamily I might mediate the response to *P*. *infestans* infection, having distinct roles in the local and systemic responses.

### Overexpression of StPP2Ac2b enhances susceptibility to *P*. *infestans*

**Fig 2:**
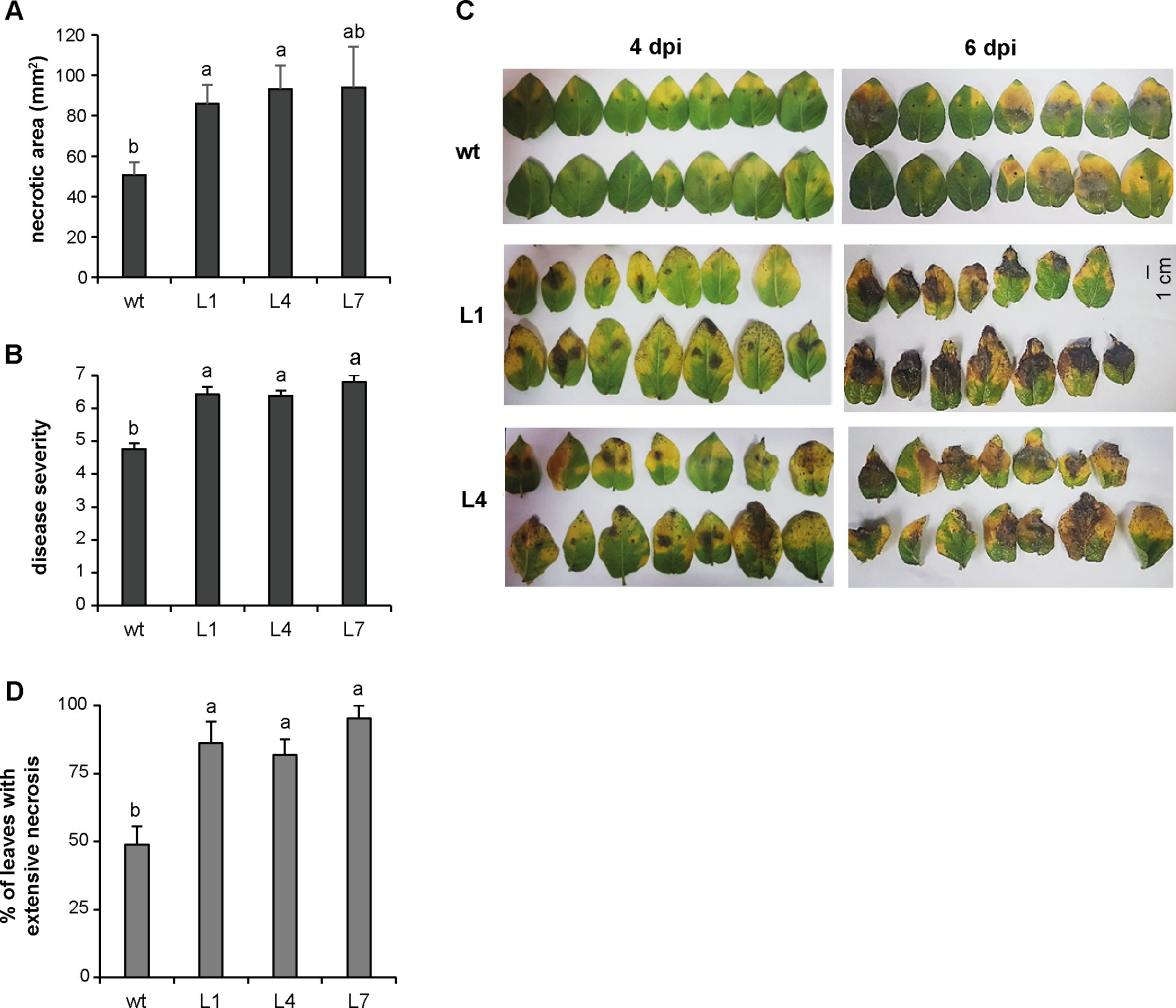
Susceptibility of StPP2Ac2b-OE plants to *P*. *infestans*. (A, B) Necrotic area and disease severity of detached leaves from wild type (wt) and PP2Ac2b-OE (L1, L4 and L7) plants four days after inoculation with *P*. *infestans*. (C) Representative images of detached leaves after four and six dpi. (D) Percentage of leaves with extensive necrosis in whole plants four dpi. Data are the mean ± SEM of of three independent experiments. Data were analyzed by one-way ANOVA followed by Bonferroni post-hoc test (α = 0.05).

To determine whether the overexpression of *StPP2Ac2b* affects the susceptibility to *P*. *infestans*, the response of StPP2Ac2b-OE plants to the infection was evaluated in detached leaves and whole plants. After inoculation of detached leaves, StPP2Ac2b-OE lines presented larger necrotic areas and increased disease severity than the wild type (Fig 2A–2C; S2 Fig). Moreover, infected StPP2Ac2b-OE leaves showed enhanced signs of senescence (Fig 2C). No symptoms of senescence were observed in non-infected detached leaves from either wild type or transgenic plants during the course of the experiment; furthermore, no differences in chlorophyll content were detected between wild type and transgenic non-infected leaves immediately after detachment or five days later (S3 Fig). When whole plants were inoculated with *P*. *infestans*, the percentage of leaves showing extensive necrosis was significantly higher in StPP2Ac2b-OE plants (Fig 2D).

### StPP2Ac2b enhances the induction of defense-related genes and senescence in response to *P*. *infestans* infection

**Fig 3:**
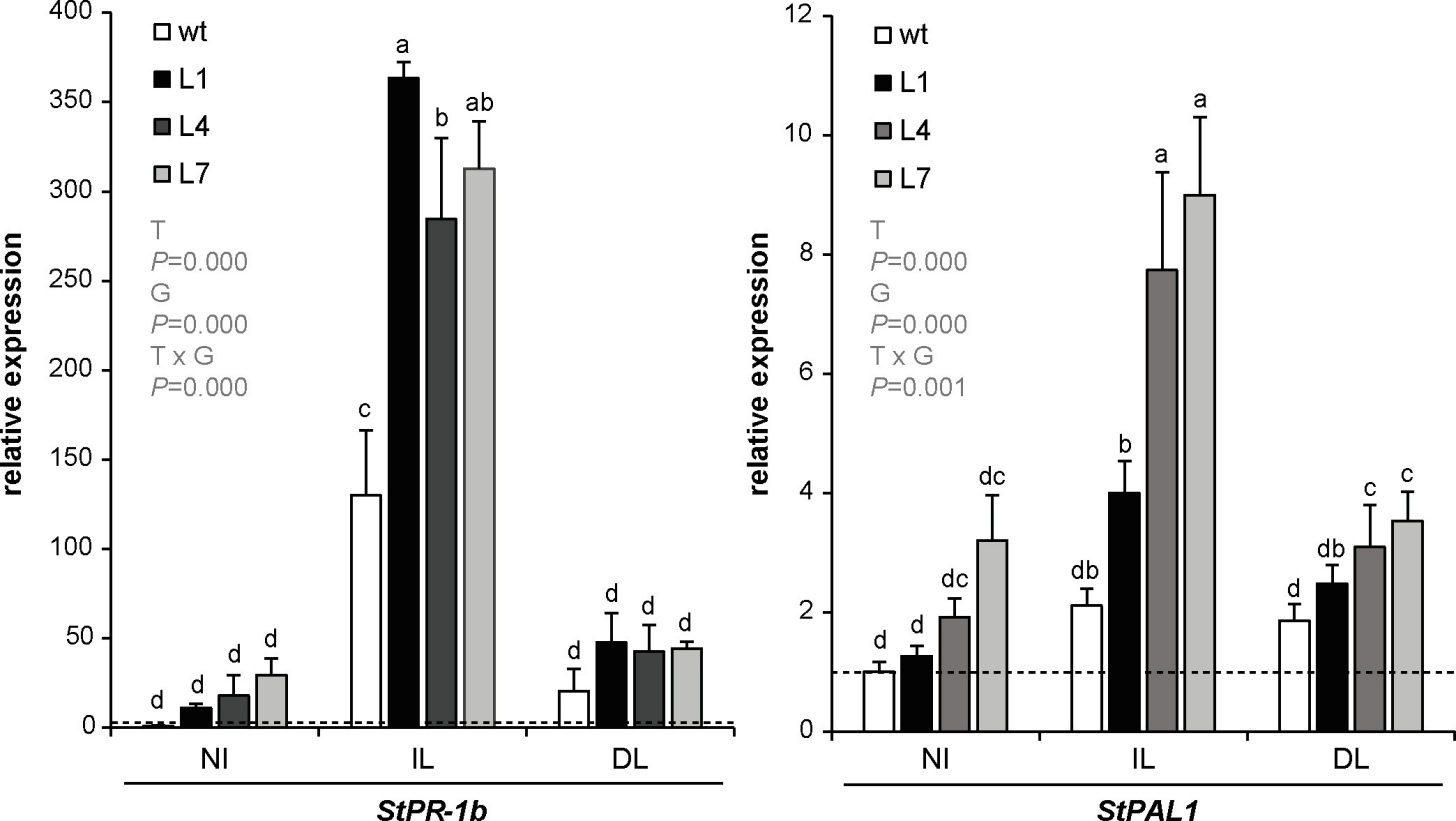
Expression of *StPR-1b* and *StPAL1* in response to *P*. *infestans* infection. Wild type (wt) and PP2Ac2b-OE (L1, L4 and L7) plants were inoculated with *P*. *infestans* as described in Materials and Methods. After four dpi, expression was determined by RT-qPCR in leaves from non-infected plants (NI), infected leaves (IL) and distal leaves (DL), relative to wt NI (dashed line). Data are the mean ± SEM of of three independent experiments. Data were analyzed by two-way ANOVA, followed by Bonferroni post-hoc test (α = 0.05) comparing the expression levels for each line (wt, L1, L4 or L7) among NI, IL and DL, and the expression levels among the different lines within each experimental condition (NI, IL or DL). *P*-values of the effect of treatment (T: NI, IL, DL), genotype (G: wt, L1, L4, L7) and their interaction are shown. The two-way ANOVA showed a significant interaction between the effects of T and G, and a significant effect of T and G on the expression levels of *StPR-1b* and *StPAL1*.

To evaluate the response of StPP2Ac2b-OE plants to *P*. *infestans* infection at the molecular level, we determined the expression of *StPR-1b* and *StPAL1*, as markers of defense response [30–32]. There were no significant differences in the expression of *StPR-1b* or *StPAL1* in non-infected leaves between StPP2Ac2b-OE lines and the wild type; however, transgenic lines showed higher levels of induction of both markers in infected leaves, and a higher induction of *StPAL1* in distal leaves (Fig 3). This result may suggest that StPP2Ac2b enhances the defense response against the pathogen.

Based on the observation that infected StPP2Ac2b-OE leaves showed enhanced signs of senescence compared with the wild type (Fig 2C), we hypothesized that a link between PP2A, senescence and susceptibility to *P*. *infestans* might exist. We analyzed the expression of senescence-promoting genes in potato RNA-seq libraries accessible from Spud DB Potato Genomic Resource (http://spuddb.uga.edu/). To this purpose, we identified 51 potato homologs of the Arabidopsis genes classified as senescence-promoting in the Leaf Senescence Database [33]. The expression profiles of these genes were obtained from RNA-seq datasets of resistant and susceptible potato genotypes, prior to infection and 1–2 days after infection with a mixture of *P*. *infestans* isolates [34]. In the absence of infection, a large number of senescence-promoting genes were up-regulated in the susceptible genotypes, compared with the resistant ones (S4A Fig, first column). In both genotypes, inoculation with the pathogen increased the expression of senescence-promoting genes (S4A Fig, second and third columns). These data suggest that *P*. *infestans* infection induces senescence in potato leaves, and that varieties with increased senescence might be more susceptible to the pathogen. Interestingly, *StPP2Ac2b* mRNA levels were higher in the susceptible genotypes than in the resistant ones, and increased after *P*. *infestans* inoculation in both genotypes (S4B Fig). This analysis supports the results obtained in this study regarding the expression of the *StPP2Ac2b* in the response to *P*. *infestans* infection, and the increased susceptibility observed in StPP2Ac2b-OE plants.

**Fig 4:**
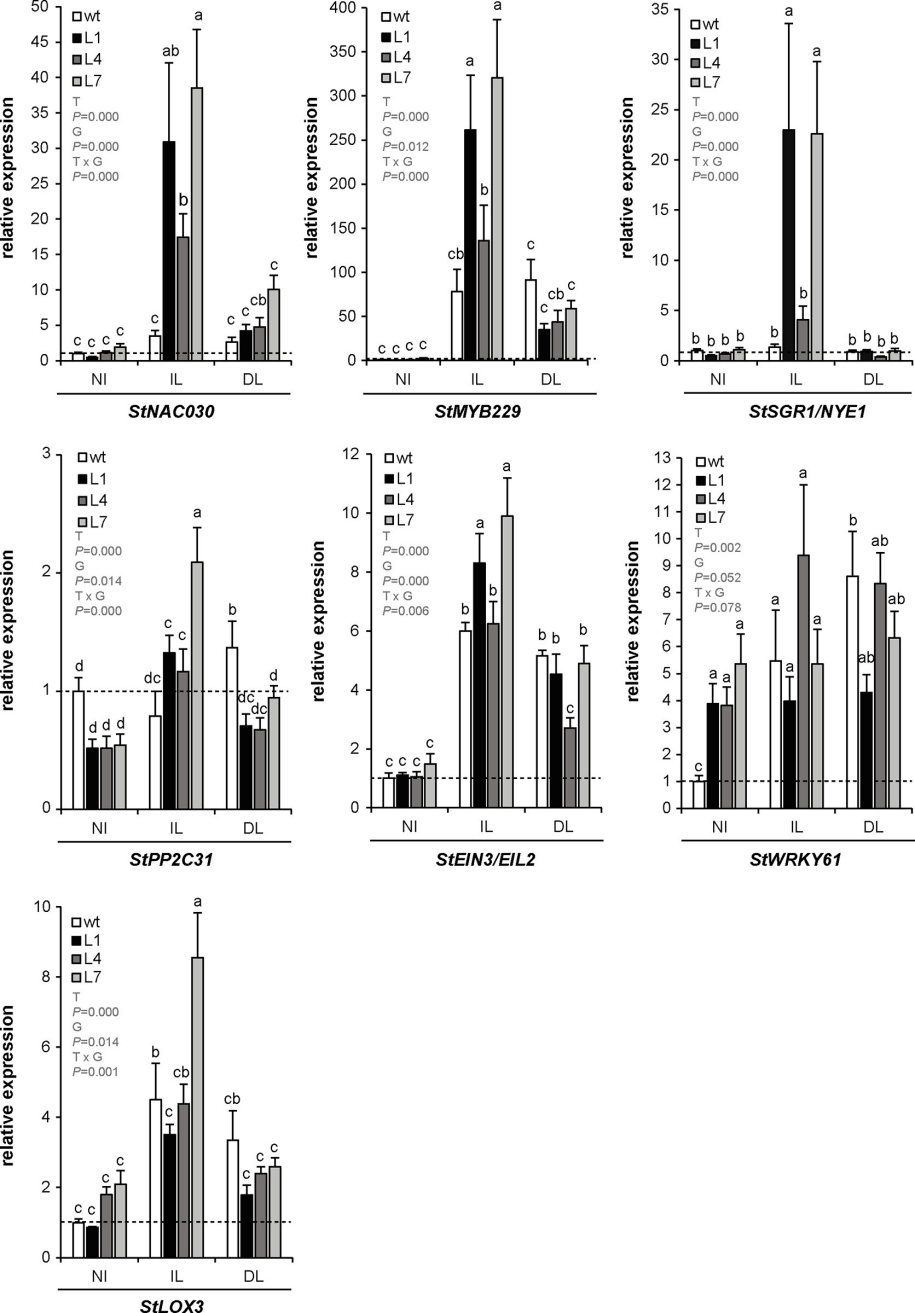
Expression of senescence marker genes in response to *P*. *infestans* infection. Wild type (wt) and PP2Ac2b-OE (L1, L4 and L7) plants were inoculated with *P*. *infestans* as described in Materials and Methods. After four dpi, expression was determined by RT-qPCR in leaves from non-infected plants (NI), infected leaves (IL) and distal leaves (DL), relative to wt NI (dashed line). Data are the mean ± SEM of three independent experiments. Data were analyzed by two-way ANOVA, followed by Bonferroni post-hoc test (α = 0.05) comparing the expression levels for each line (wt, L1, L4 or L7) among NI, IL and DL, and the expression levels among the different lines within each experimental condition (NI, IL or DL). *P*-values of the effect of treatment (T: NI, IL, DL), genotype (G: wt, L1, L4, L7) and their interaction are shown. The two-way ANOVA revealed a significant interaction between the effects of T and G, and a significant effect of T and G on the expression levels of all the senescence marker genes analyzed, except for *StWRKY61* (non-significant interaction; significant effect of T; non-significant effect of G).

Next, leaf senescence in response to *P*. *infestans* infection was evaluated in StPP2Ac2b-OE lines. Whole plants were used, since it has been demonstrated that detached leaves become naturally more senescent and more susceptible to hemibiotrophic pathogens [35]. *StNAC030*, *StMYB229*, *StSGR1/NYE1*, *StPP2C31*, *StEIN3/EIL2*, *StWRKY61* and *StLOX3* were selected as markers of senescence (S1 Table). These genes are homologs of Arabidopsis senescence-promoting genes, and showed a clear induction in response to *P*. *infestans* according to the RNA-seq datasets mentioned above. As shown in Fig 4, there were no significant differences in the mRNA levels of *StNAC030*, *StMYB229*, *StSGR1/NYE1* and *StPP2C31* in non-infected leaves between wild type and StPP2Ac2b-OE lines; the expression of these genes was induced after *P*. *infestans* inoculation in transgenic lines, but not in the wild type. The mRNA levels of *StEIN3/EIL2* were significantly increased after pathogen infection in both wild type and StPP2Ac2b-OE plants, but this induction was more marked in transgenic lines. *StWRKY61* was upregulated in non-infected leaves from transgenic plants, but no further induction was observed in infected leaves; in wild type plants, *StWRKY61* expression increased after *P*. *infestans* inoculation. There were no significant differences in the mRNA levels of *StLOX3* in non-infected leaves between wild type and StPP2Ac2b-OE lines; *StLOX3* expression was induced in response to *P*. *infestans* infection in wild type and L7 leaves, but the induction in line L7 was more marked. No pathogen-triggered induction of *StLOX3* was observed for lines L1 and L4. Overall, these results indicate that *P*. *infestans* infection induces the expression of senescence-promoting genes in potato plants and this response is more pronounced in StPP2Ac2b-OE lines. Interestingly, *StPP2C31*, *StEIN3/EIL2*, and *StWRKY61* were induced in distal leaves of infected plants (Fig 4), suggesting a systemic senescence response to the pathogen.

**Fig 5:**
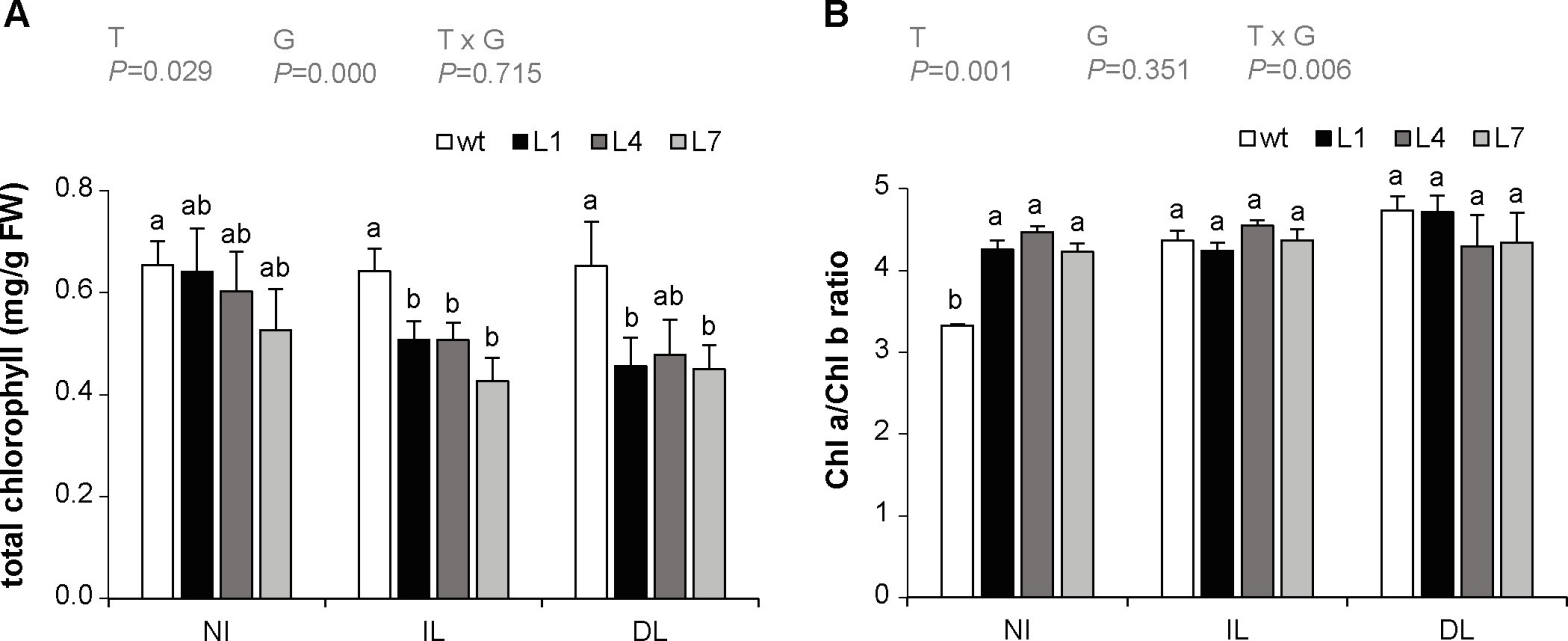
Changes in chlorophyll content in response to *P*. *infestans* infection. Wild type (wt) and PP2Ac2b-OE (L1, L4 and L7) plants were inoculated with *P*. *infestans* as described in Materials and Methods. Leaves from non-infected plants (NI), infected leaves (IL) and distal leaves (DL) were collected after four dpi for chlorophyll measurement. (A) Total chlorophyll content. (B) Ratio between chlorophyll a and chlorophyll b. Data are the mean ± SEM of of three independent experiments. Data were analyzed by two-way ANOVA, followed by Bonferroni post-hoc test (α = 0.05) comparing the total chlorophyll content or the Chl a/Chl b ratio for each line (wt, L1, L4 or L7) among NI, IL and DL, and the total chlorophyll content or the Chl a/Chl b ratio among the different lines within each experimental condition (NI, IL or DL). *P*-values of the effect of treatment (T: NI, IL, DL), genotype (G: wt, L1, L4, L7) and their interaction are shown. The two-way ANOVA showed a significant interaction between the effects of T and G on the Chl a/Chl b ratio (B). There was a significant effect of T on total chlorophyll and the Chl a/Chl b ratio (A; B), and a significant effect of G on total chlorophyll (A).

Chlorophyll content was also determined to evaluate senescence in response to the pathogen. There were no significant differences between wild type and StPP2Ac2b-OE lines in the total chlorophyll content of non-infected leaves, but in infected and distal leaves, transgenic lines showed lower levels of chlorophyll than the wild type (Fig 5A). This result is in agreement with the enhanced signs of senescence observed in StPP2Ac2b-OE leaves inoculated with *P*. *infestans* (Fig 2C). In non-infected leaves, the ratio between chlorophyll a and chlorophyll b was higher in StPP2Ac2b-OE lines than in the wild type. This ratio increased in wild type leaves after inoculation, reaching values similar to those of non-infected transgenic leaves (Fig 5B).

### StPP2Ac2b accelerates senescence in potato plants

**Fig 6:**
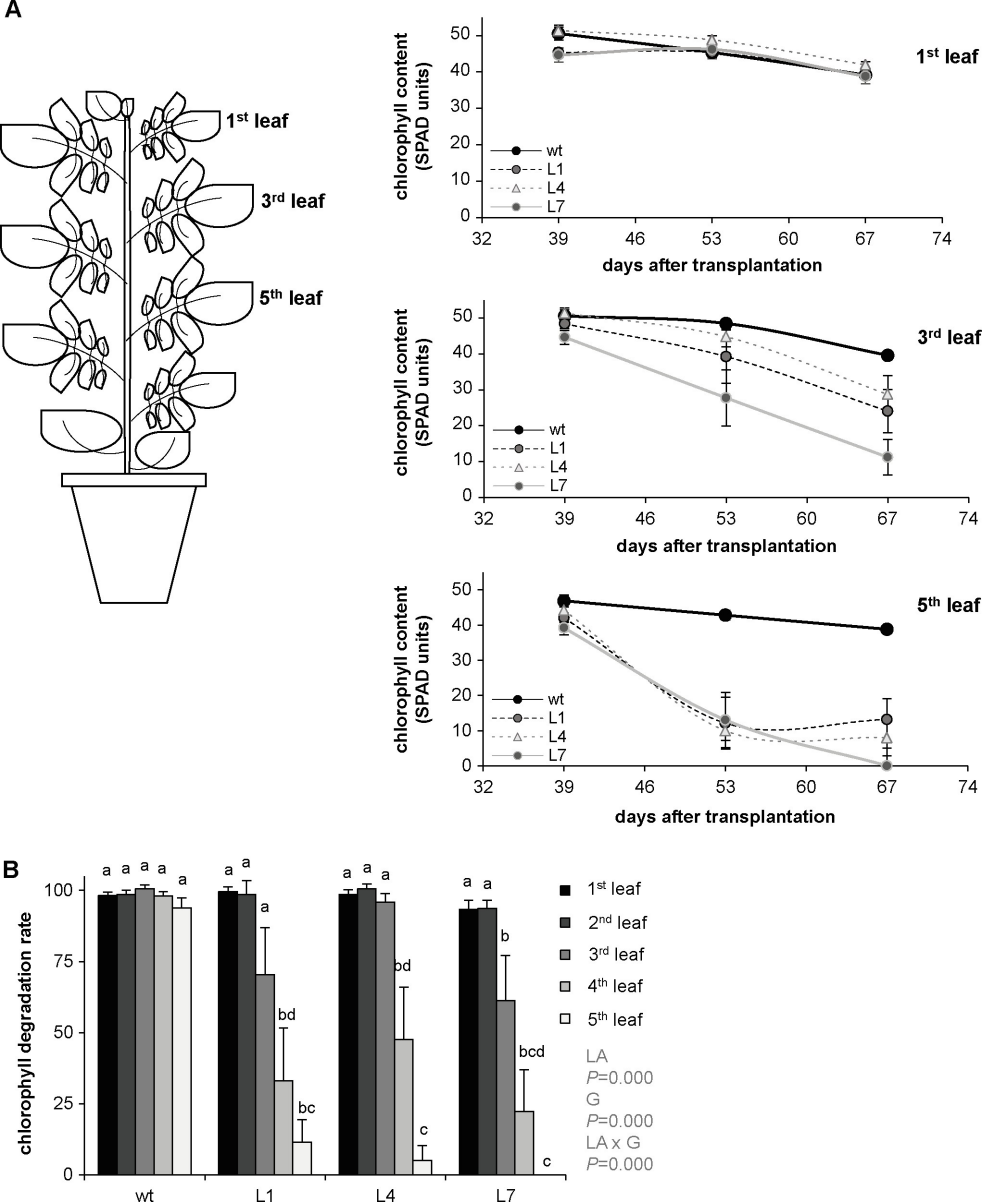
Leaf senescence in StPP2Ac2b-OE plants. Wild type (wt) and PP2Ac2b-OE (L1, L4 and L7) plants transferred to soil ex vitro were cultivated in a growth chamber. After different times, chlorophyll content was determined. (A) Chlorophyll content in the first (upper), third and fifth leaf, 39, 53 and 67 days after transplantation to soil (statistical analysis is shown in S6 Fig). (B) Chlorophyll degradation rate, calculated as 100*(chlorophyll content 53 days after transplantation/chlorophyll content 39 days after transplantation). Data are the mean ± SEM of four to eight plants per line (repeated three times independently with similar results). Data were analyzed by two-way ANOVA, followed by Bonferroni post-hoc test (α = 0.05) comparing the degradation rate for each leaf (first, second, third, fourth or fifth) between wt, L1, L4 and L7, and the degradation rate among the different leaves for each line (wt, L1, L4 or L7). *P*-values of the effect of leaf age (LA: first, second, third, fourth, fifth leaf), genotype (G: wt, L1, L4, L7) and their interaction are shown. The two-way ANOVA revealed a significant interaction between the effects of LA and G, and a significant effect of LA and G on the on the chlorophyll degradation rate.

Besides the senescence induced by the pathogen, we evaluated the developmental senescence in StPP2Ac2b-OE plants by measuring the chlorophyll content in the first (upper), second, third, fourth and fifth leaf at different times of growth in soil after ex vitro transfer (Fig 6). The chlorophyll content of the first leaf was similar in StPP2Ac2b-OE and wild type plants after 39, 53 and 67 days, and did not change significantly over time in either wild type or transgenic lines, except for a slight decrease after 67 days (Fig 6A; S6 Fig). The chlorophyll content of the third and fifth leaf was similar in StPP2Ac2b-OE and wild type plants after 39 days, but decreased significantly in transgenic lines after 53 and 67 days, while remained practically unchanged in the wild type (Fig 6A; S6 Fig). The reduction in chlorophyll content over time in StPP2Ac2b-OE lines was more pronounced in the fifth leaf than in the third leaf. Accordingly, the chlorophyll degradation rate was positively correlated with leaf age in transgenic plants; it was more marked in the fourth and fifth leaf than in the third leaf (Fig 6B). No chlorophyll degradation was observed in the youngest leaves (first and second leaf) of StPP2Ac2b-OE lines, or in any leaf of wild type plants (Fig 6B). The lower leaves of two-month old transgenic plants showed more signs of senescence and abscission than the wild type, but the upper leaves remained green and healthy (S5 Fig). These results indicate that StPP2Ac2b accelerates developmental senescence once this process is naturally initiated, however, it seems that StPP2Ac2b per se is not able to induce senescence, since in young leaves, there are no differences in the chlorophyll content nor visible signs of senescence between transgenic and wild type plants.

We noticed that after chlorophyll determination using the SPAD meter, StPP2Ac2b-OE leaves developed a chlorotic lesion that turned necrotic around the site of measurement, while this lesion was absent in wild type leaves (S7A Fig). This effect was accompanied by a decrease in chlorophyll content (S7B Fig). This observation may suggest that StPP2Ac2b, in addition to accelerating developmental senescence, promotes localized senescence and cell death after mechanical damage in the tissue surrounding the point of injury. This process, described and characterized previously, is aimed to prevent the spread of cell death after mechanical wounding [36, 37].

## Discussion

The role of PP2A in the response to fungal pathogens has been investigated in several plant species, however, little is known about the function of this phosphatase in plant defense against oomycete pathogens, which have a great impact on humankind, due their permanent threat to commercial crops and destructive effects on native plants [38]. The aim of this study was to evaluate the role of StPP2Ac2b in the defense response to *P*. *infestans*. Inoculation of potato plants with the pathogen induces the expression of *StPP2Ac2b* in infected and distal leaves (Fig 1), suggesting that this catalytic subunit might be involved in the molecular response to *P*. *infestans*.

Overexpression of StPP2Ac2b increases the susceptibility to *P*. *infestans* (Fig 2). This finding is in agreement with previous reports by He et al. [16], Zhu et al. [17] and Trotta et al. [20] which show that silencing or knockout of PP2A catalytic or regulatory subunits confers resistance to different pathogens in *N*. *benthamiana*, wheat and Arabidopsis. These reports, together with the present study, provide strong genetic evidence indicating a negative effect of PP2A on pathogen resistance. However, the results recently obtained by Chen et al. [19] and Lin et al. [18] show a positive role of PP2A in protecting *N*. *benthamiana* and rice plants against pathogens. Therefore, PP2A might have different effects on resistance or susceptibility, which probably depend on the specific plant-pathogen interaction.

It has been previously shown that PP2A can regulate the expression of defense-related genes either positively [18] or negatively [16, 17, 21], and this effect is correlated with enhanced or attenuated resistance, respectively. Conversely, the increased susceptibility of StPP2Ac2b-OE plants to late blight cannot be explained by the regulation of the defense response at the molecular level, since StPP2Ac2b enhances the expression of defense-related genes in response to *P*. *infestans* infection (Fig 3). This finding suggests that, for the specific potato cultivar used in this study, a different PP2A-mediated regulation of the plant response to *P*. *infestans* might exist. However, it cannot be excluded that for other potato cultivars the correlation between plant susceptibility and defense-related genes expression is opposite to the one here observed.

Inoculation with *P*. *infestans* induces senescence in both wild type and StPP2Ac2b-OE plants, but this response is more pronounced in transgenic lines (Figs 2C, 4 and 5). StPP2Ac2b-OE plants also exhibit enhanced developmental senescence (Fig 6). These results suggest that senescence induction, mediated by StPP2Ac2b, might be part of the pathogenicity mechanism of *P*. *infestans*. Supporting this hypothesis, analysis of RNA-seq datasets revealed that potato genotypes susceptible to *P*. *infestans* show higher expression levels of senescence-promoting genes than resistant genotypes, and that *P*. *infestans* infection induces senescence-promoting genes in both genotypes (S4A Fig). Moreover, *StPP2Ac2b* mRNA levels are higher in the susceptible genotypes (S4B Fig).

Increasing evidence reveals a correlation between senescence and pathogen resistance or susceptibility [39, 40]. Dhar et al. [41] showed that SAG13 (SENESCENCE ASSOCIATED GENE 13) promotes leaf senescence in Arabidopsis, and acts as a negative regulator of defense against biothophic pathogens, but enhances resistance to *B*. *cinerea*. In agreement with these findings, Durian et al. [21] showed that PP2A-B´γ delays senescence and prevents the defense response against *B*. *cinerea*. Barth et al. [42] and Guo et al. [43] found a positive correlation between senescence and resistance to the hemibiotrophic pathogens *P*. *syringae* and *Peronospora parasitica* in Arabidopsis. According to our results, there is a negative correlation between senescence and resistance to *P*. *infestans* in potato plants. Therefore, the correlation between senescence and resistance may be positive or negative depending on the particular interaction between the host and the pathogen.

It is important to note that StPP2Ac2b-OE lines L1 and L7 show a reduction in plant size, compared with the wild type (S1 Fig). However, in whole plants, the response to the pathogen in these lines is similar to that observed in L4 (Fig 2D), which is comparable in size to the wild type (S1 Fig). At the molecular level, there are no clear differences among the three transgenic lines in the defense response or the pathogen-triggered senescence (Figs 3, 4 and 5). Therefore, the phenotype observed in StPP2Ac2b-OE plants is more likely due to a specific regulation of the response to the pathogen by StPP2Ac2b than an altered growth-defense trade off balance. Moreover, in the infection experiments with detached leaves, the leaf size of the three StPP2Ac2b-OE lines was similar to that of the wild type, and there were no differences in the susceptibility among the transgenic lines (Fig 2A–2C; S2 Fig), suggesting that that the susceptibility to *P*. *infestans* is not affected by leaf size in StPP2Ac2b-OE plants.

The role of PP2A in senescence remains largely unknown. Our results show that StPP2Ac2b is a positive regulator of developmental senescence (Fig 6), pathogen-triggered senescence (Figs 2C, 4 and 5) and localized cell death induced by mechanical damage (S7 Fig). In contrast, as mentioned before, it was demonstrated that the regulatory subunit PP2A-B´γ inhibits developmental senescence in Arabidopsis [21]. These findings are not contradictory, since each regulatory B subunit may bind to several different combinations of PP2A A-C dimers, thus trimeric holoenzymes with different B subunits can modulate cellular responses in both redundant and opposing ways. Determining the composition of the PP2A holoenzymes that regulate senescence and defense responses will shed light on the molecular mechanisms that control these important processes.

In conclusion, our findings indicate that StPP2Ac2b positively regulates developmental and pathogen-induced senescence in potato plants, and reveal that *P*. *infestans* infection promotes senescence, possibly through induction of *StPP2Ac2b* expression.

## Supporting information

S1 Fig Wild type and StPP2Ac2b-OE plants before and after inoculation with *P*. *infestans*. Representative image of the plants used in the infection experiments before inoculation (non-infected, NI) and four dpi.
(PDF)

S2 Fig Susceptibility of StPP2Ac2b-OE plants to *P*. *infestans*. Percentage of leaf area diseased in detached leaves from wild type (wt) and PP2Ac2b-OE (L1, L4 and L7) plants four days after inoculation with the pathogen. The percentage of leaf area diseased was determined as the percentage of the leaf area presenting symptoms (necrosis, chlorosis, presence of mycelium). Data are the mean ± SEM of of three independent experiments. Data were analyzed by one-way ANOVA followed by Bonferroni post-hoc test (α = 0.05).
(PDF)

S3 Fig Chlorophyll content in detached leaves. Chlorophyll content and representative image of non-infected detached leaves from wild type (wt) and PP2Ac2b-OE (L1, L4 and L7) plants, immediately after detachment (day 0) and five days after detachment (day 5). Data are the mean ± SEM of of three independent experiments. Data were analyzed by two-way ANOVA, followed by Bonferroni post-hoc test (α = 0.05) comparing the chlorophyll content for each line (wt, L1, L4 or L7) between day 0 and day 5, and the chlorophyll content among the different lines within each time point (day 0 or day 5). *P*-values of the effect of time since leaf detachment (T: day 0, day 5), genotype (G: wt, L1, L4, L7) and their interaction are shown.
(PDF)

S4 Fig Expression profiles of senescence-promoting genes and PP2Ac isoforms in resistant and susceptible potato genotypes. Heat maps visualization of RNA-seq data available at http://spuddb.uga.edu/, obtained from resistant and susceptible potato genotypes (pool of leaves from of eight heterozygous genotypes), prior to *P*. *infestans* infection and after 1–2 dpi (NCBI accession numbers: SRX1257734, SRX1257735, SRX1257736, SRX1257737). (A) Expression profiles of senescence promoting genes; transcript IDs are shown in S2 Table. (B) Expression profiles of PP2Ac isoforms. Differential expression was determined as log2 fold change; first column: susceptible genotypes vs. resistant genotypes; second column: resistant genotypes, *P*. *infestans* inoculated (Pi) vs. control (non-infected); third column: susceptible genotypes, *P*. *infestans* inoculated vs. control. Heat maps were constructed using the Heatmapper web server (http://www.heatmapper.ca/).
(PDF)

S5 Fig Leaf senescence in StPP2Ac2b-OE plants. Representative image of wild type (wt) and PP2Ac2b-OE (L1, L4 and L7) plants grown in soil for two months.
(PDF)

S6 Fig Statistical analysis of Fig 6A. Chlorophyll content in the first (upper), third and fifth leaf, 39, 53 and 67 days after transplantation to soil. Data are the mean ± SEM of four to eight plants per line (repeated three times independently with similar results). Data were analyzed by two-way ANOVA, followed by Bonferroni post-hoc test (α = 0.05) comparing the chlorophyll content in each line (wt, L1, L4 or L7) between 39, 53 and 67 days, and the chlorophyll content among the different lines within each time point (39, 53 or 67 days). *P*-values of the effect of time after transplantation (T: 39, 53, 67 days), genotype (G: wt, L1, L4, L7) and their interaction are shown. The two-way ANOVA showed a significant interaction between the effects of T and G on chlorophyll content of the fifth leaf, and a significant effect of T and G on chlorophyll content of the first, third and fifth leaf.
(PDF)

S7 Fig StPP2Ac2b promotes localized cell death after mechanical damage. Wild type (wt) and PP2Ac2b-OE (L1, L4 and L7) plants transferred to soil ex vitro were cultivated in a growth chamber for four weeks. The first and second fully expanded leaves were subjected to mechanical damage using the SPAD meter to determine chlorophyll content in two different points per leaf. Five days later, the measurement was repeated. (A) Representative image of the first leaf of wt and L1 subjected to mechanical damage (five days after the first SPAD measurement). (B) Relative chlorophyll content of the first and second leaves (pooled together) five days after mechanical damage. Data are the mean ± SEM of 10 plants per line (repeated three times independently with similar results). Data were analyzed by two-way ANOVA, followed by Bonferroni post-hoc test (α = 0.05) comparing the relative chlorophyll content for each line (wt, L1, L4 or L7) between control and damage, and the relative chlorophyll content among the different lines within each experimental condition (control or damage). *P*-values of the effect of treatment (T: control, damage), genotype (G: wt, L1, L4, L7) and their interaction are shown. The two-way ANOVA showed a significant effect of T on the relative chlorophyll content.
(PDF)

S1 Table Primers used for RT-qPCR analysis. Forward and reverse primers used for RT-qPCR analysis. Information of the senescence marker genes used in this study is also shown. Sequence ID (Potato Genomics Resource; http://spuddb.uga.edu/index.shtml); *S*. *lycopersicum* homologs (Sol Genomics Network; https://solgenomics.net/). The primers sequences were obtained from the reports indicated as reference.
(PDF)

S2 Table Transcript IDs of senescence-promoting genes. Transcript IDs of senescence-promoting genes used to construct the heat map of S4 Fig.
(PDF)
