# Supplementary figures and images for: The protein phosphatase 2A catalytic subunit StPP2Ac2b enhances susceptibility to Phytophthora infestans and senescence in potato
Source: PLoS One. 2022 Oct 10;17(10):e0275844. doi: 10.1371/journal.pone.0275844 (PMC9550054; doi:10.1371/journal.pone.0275844)

NI

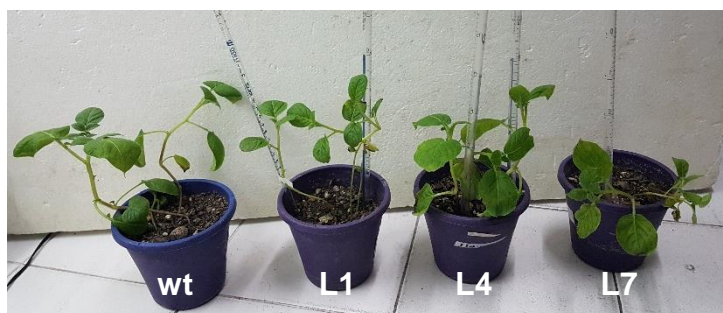

4 dpi

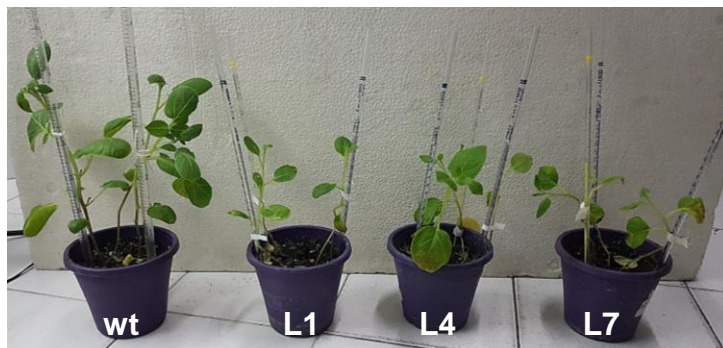

Supplement: S1 Fig — Representative image of the plants used in the infection experiments before inoculation (non-infected, NI) and four dpi. (PDF) [file pone.0275844.s001.pdf]

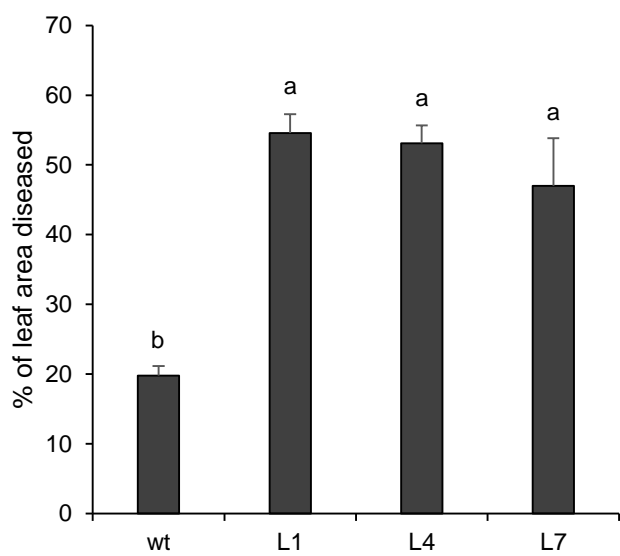

Supplement: S2 Fig — Percentage of leaf area diseased in detached leaves from wild type (wt) and PP2Ac2b-OE (L1, L4 and L7) plants four days after inoculation with the pathogen. The percentage of leaf area diseased was determined as the percentage of the leaf area presenting symptoms (necrosis, chlorosis, presence of mycelium). Data are the mean ± SEM of of three independent experiments. Data were analyzed by one-way ANOVA followed by Bonferroni post-hoc test (α = 0.05). (PDF) [file pone.0275844.s002.pdf]

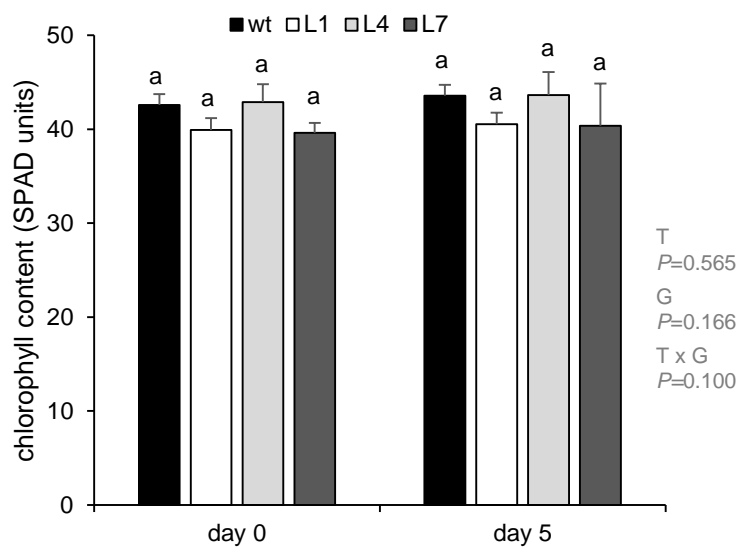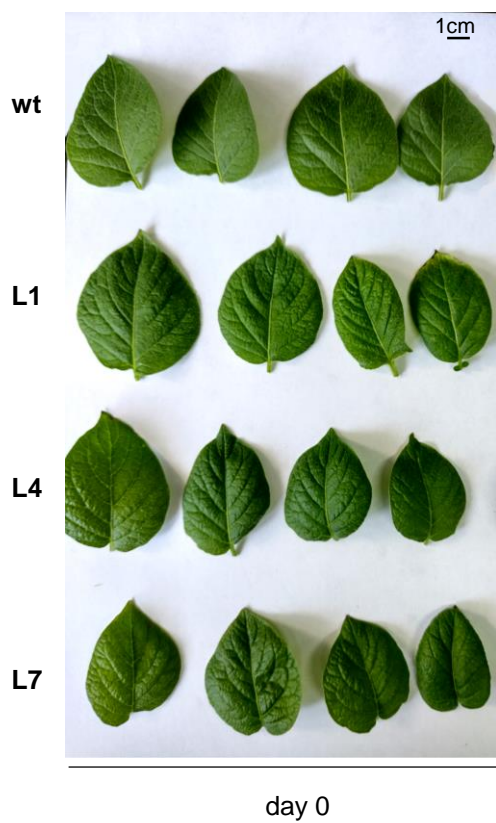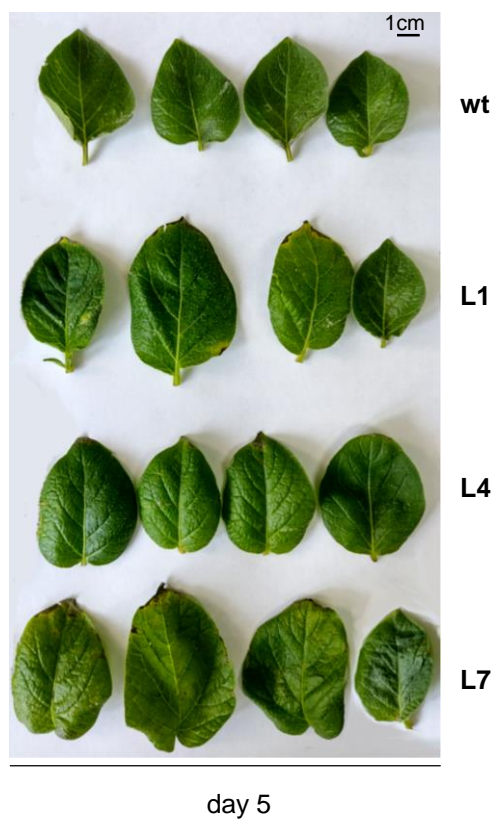

Supplement: S3 Fig — Chlorophyll content and representative image of non-infected detached leaves from wild type (wt) and PP2Ac2b-OE (L1, L4 and L7) plants, immediately after detachment (day 0) and five days after detachment (day 5). Data are the mean ± SEM of of three independent experiments. Data were analyzed by two-way ANOVA, followed by Bonferroni post-hoc test (α = 0.05) comparing the chlorophyll content for each line (wt, L1, L4 or L7) between day 0 and day 5, and the chlorophyll content among the different lines within each time point (day 0 or day 5). P-values of the effect of time since leaf detachment (T: day 0, day 5), genotype (G: wt, L1, L4, L7) and their interaction are shown. (PDF) [file pone.0275844.s003.pdf]

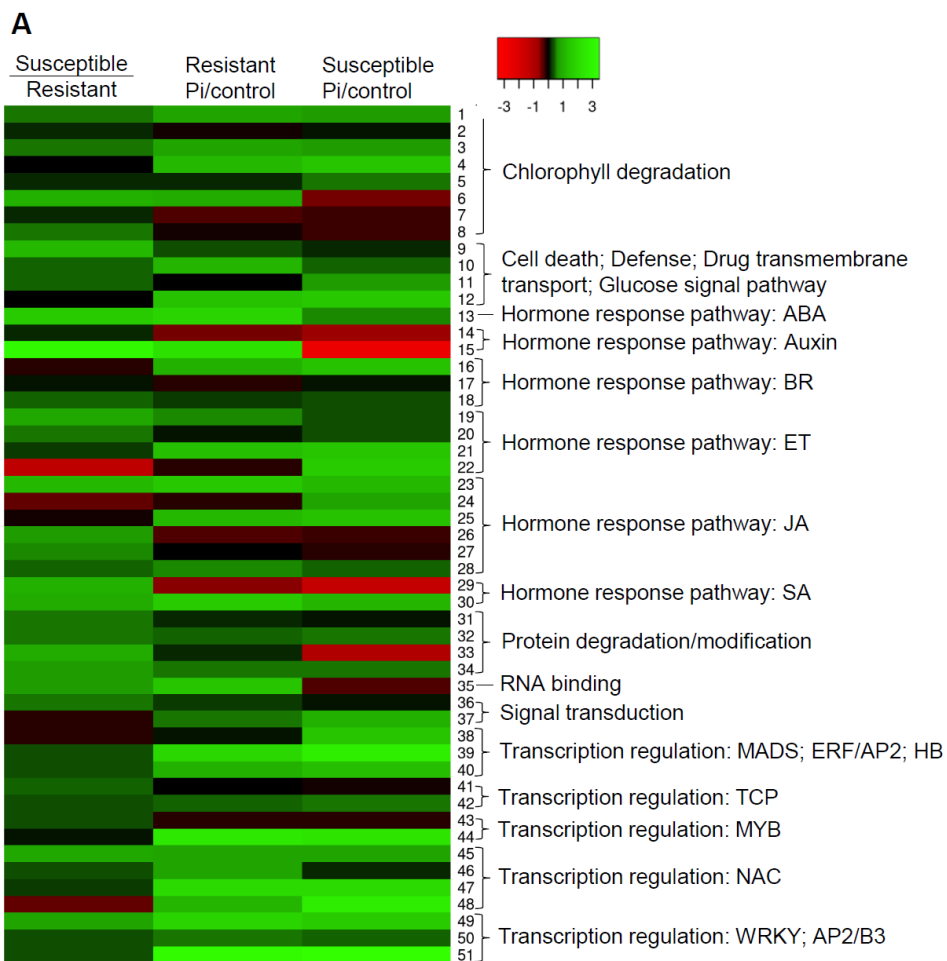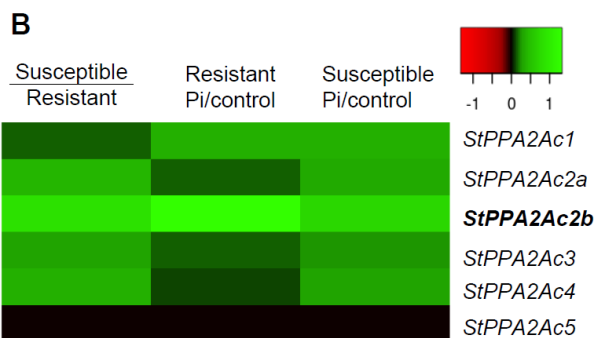

Supplement: S4 Fig — Heat maps visualization of RNA-seq data available at http://spuddb.uga.edu/, obtained from resistant and susceptible potato genotypes (pool of leaves from of eight heterozygous genotypes), prior to P. infestans infection and after 1–2 dpi (NCBI accession numbers: SRX1257734, SRX1257735, SRX1257736, SRX1257737). (A) Expression profiles of senescence promoting genes; transcript IDs are shown in S2 Table. (B) Expression profiles of PP2Ac isoforms. Differential expression was determined as log2 fold change; first column: susceptible genotypes vs. resistant genotypes; second column: resistant genotypes, P. infestans inoculated (Pi) vs. control (non-infected); third column: susceptible genotypes, P. infestans inoculated vs. control. Heat maps were constructed using the Heatmapper web server (http://www.heatmapper.ca/). (PDF) [file pone.0275844.s004.pdf]

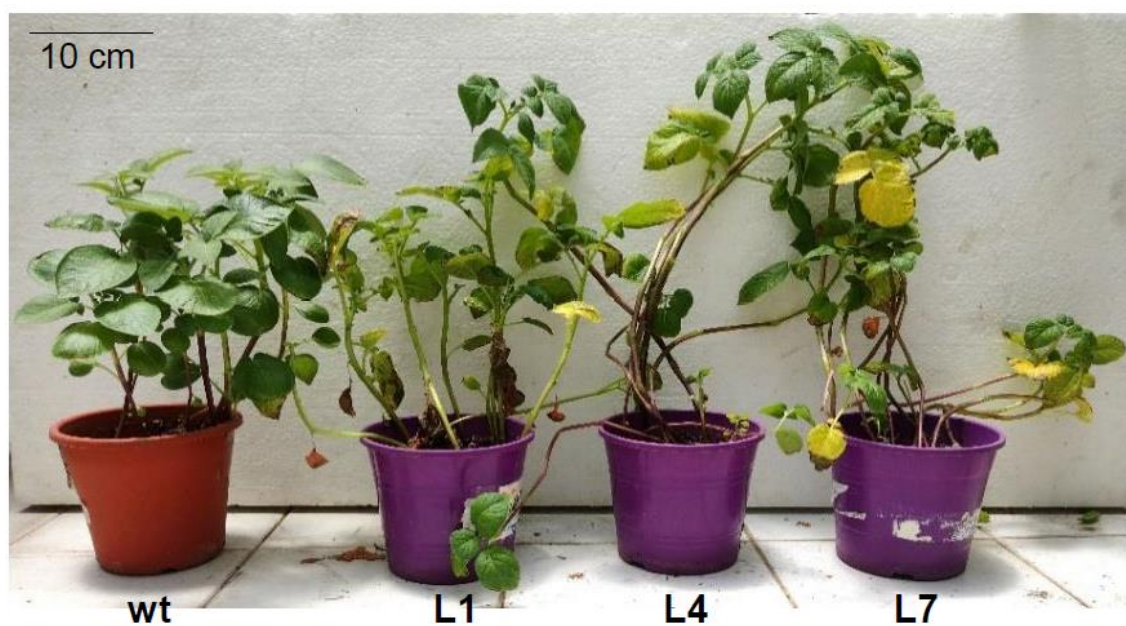

Supplement: S5 Fig — Representative image of wild type (wt) and PP2Ac2b-OE (L1, L4 and L7) plants grown in soil for two months. (PDF) [file pone.0275844.s005.pdf]

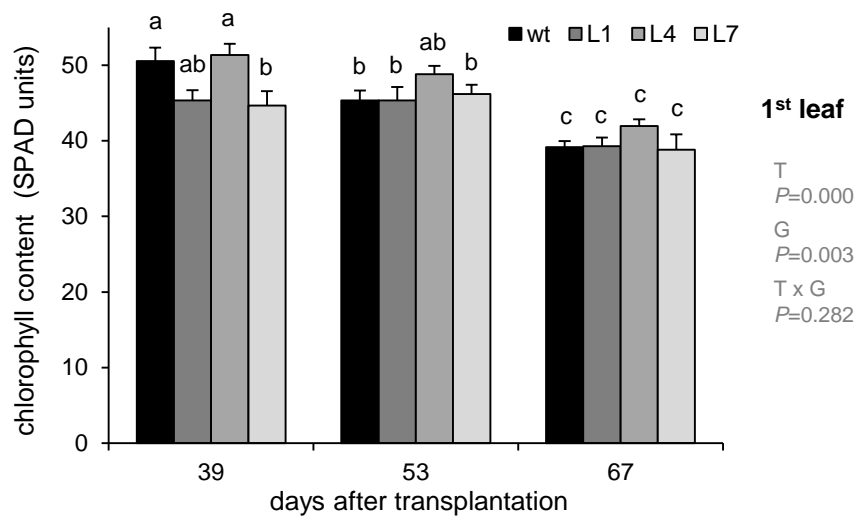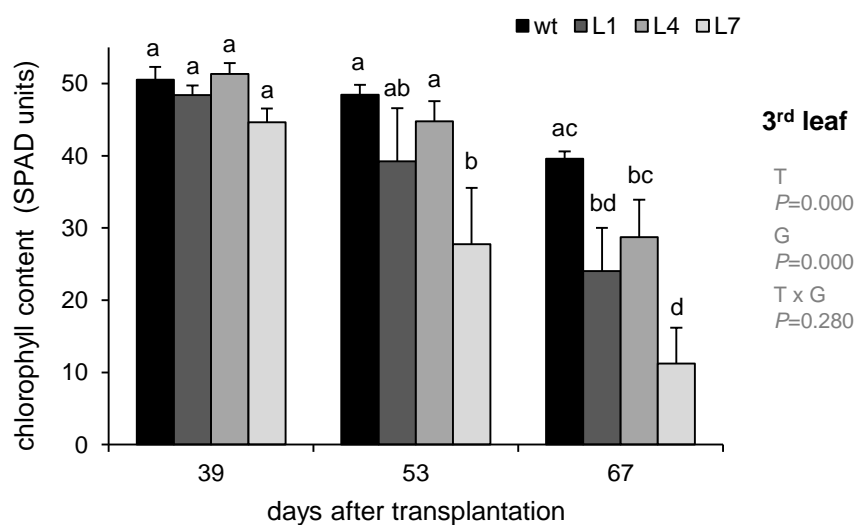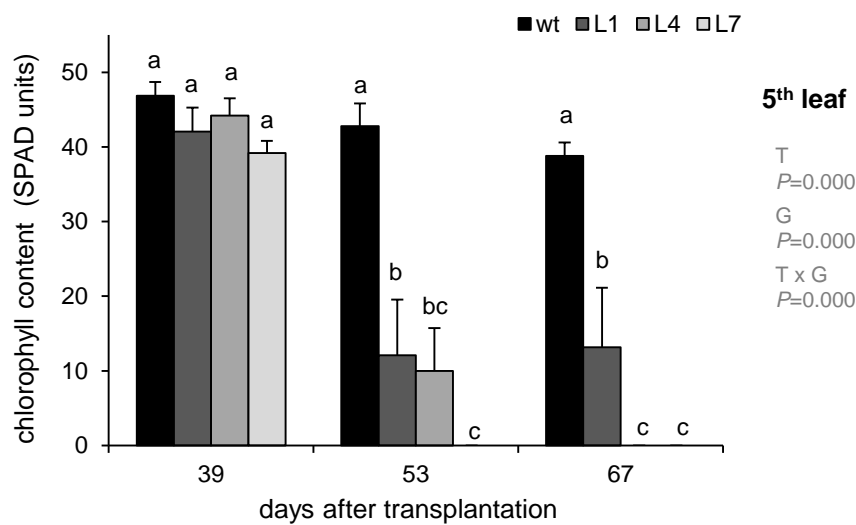

Supplement: S6 Fig — Chlorophyll content in the first (upper), third and fifth leaf, 39, 53 and 67 days after transplantation to soil. Data are the mean ± SEM of four to eight plants per line (repeated three times independently with similar results). Data were analyzed by two-way ANOVA, followed by Bonferroni post-hoc test (α = 0.05) comparing the chlorophyll content in each line (wt, L1, L4 or L7) between 39, 53 and 67 days, and the chlorophyll content among the different lines within each time point (39, 53 or 67 days). P-values of the effect of time after transplantation (T: 39, 53, 67 days), genotype (G: wt, L1, L4, L7) and their interaction are shown. The two-way ANOVA showed a significant interaction between the effects of T and G on chlorophyll content of the fifth leaf, and a significant effect of T and G on chlorophyll content of the first, third and fifth leaf. (PDF) [file pone.0275844.s006.pdf]

**A**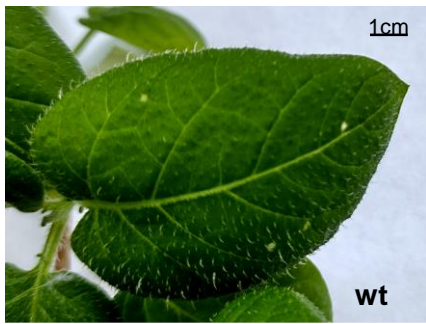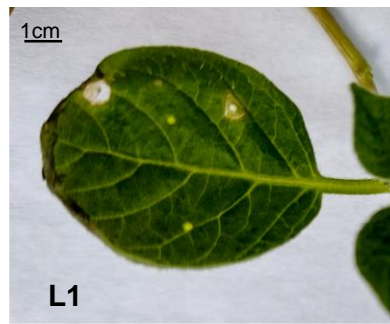**B**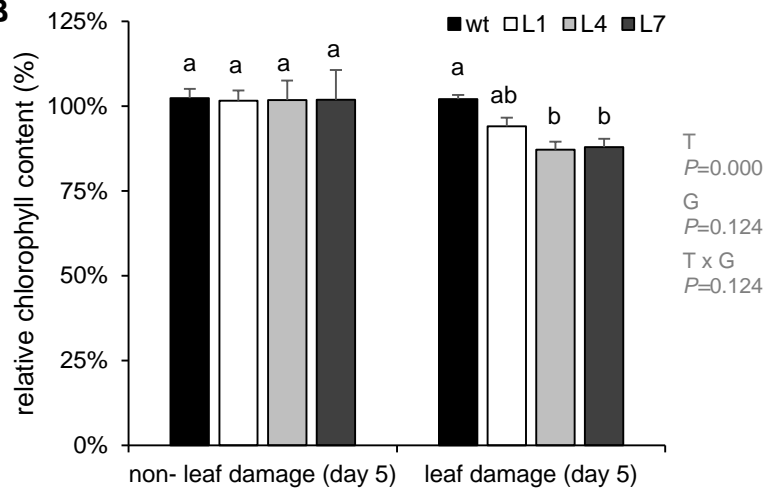

Supplement: S7 Fig — Wild type (wt) and PP2Ac2b-OE (L1, L4 and L7) plants transferred to soil ex vitro were cultivated in a growth chamber for four weeks. The first and second fully expanded leaves were subjected to mechanical damage using the SPAD meter to determine chlorophyll content in two different points per leaf. Five days later, the measurement was repeated. (A) Representative image of the first leaf of wt and L1 subjected to mechanical damage (five days after the first SPAD measurement). (B) Relative chlorophyll content of the first and second leaves (pooled together) five days after mechanical damage. Data are the mean ± SEM of 10 plants per line (repeated three times independently with similar results). Data were analyzed by two-way ANOVA, followed by Bonferroni post-hoc test (α = 0.05) comparing the relative chlorophyll content for each line (wt, L1, L4 or L7) between control and damage, and the relative chlorophyll content among the different lines within each experimental condition (control or damage). P-values of the effect of treatment (T: control, damage), genotype (G: wt, L1, L4, L7) and their interaction are shown. The two-way ANOVA showed a significant effect of T on the relative chlorophyll content. (PDF) [file pone.0275844.s007.pdf]
