# Supplementary material for: The protein phosphatase 2A catalytic subunit StPP2Ac2b enhances susceptibility to Phytophthora infestans and senescence in potato
Source: PLoS One. 2022 Oct 10;17(10):e0275844. doi: 10.1371/journal.pone.0275844 (PMC9550054; doi:10.1371/journal.pone.0275844)
Supplement: S1 Table — Forward and reverse primers used for RT-qPCR analysis. Information of the senescence marker genes used in this study is also shown. Sequence ID (Potato Genomics Resource; http://spuddb.uga.edu/index.shtml); S. lycopersicum homologs (Sol Genomics Network; https://solgenomics.net/). The primers sequences were obtained from the reports indicated as reference. (PDF) [file pone.0275844.s008.pdf]

	Forward primer	Reverse Primer
EF1-α	ATTGGAAACGGATATGCTCCA	TCCTTACCTGAACGCCTGTCA
StPP2Ac2b	GAGAGTTGAGAAGAGGCACTG	CTATGGAATACCAAATATACAGAC
StPP2Ac1	CCGAATACTTGAGCTGACTG	ATTACAAATCACAATGGACC
StPP2Ac2a	TTCCAATATCTGCGCTTG	GAACAGAATGTACCATGTTGC
StPR-1b	GCCCAAAATTCACCCCAAGAC	CTGCACCGGAATGAATCAAGT
StPAL1	GGTGTTACTACTGGATTTGGTGC	CCTAACAAGCATAGCTGCCC
StNAC030	CAAGATCCTGCAACTCTGAGG	CCTGTTTCTTGGGATACAGTGA
StMYB229	CTCGTTCTGCAGGTTTAAAGAG	GTGCAATTTTCGACCACCTATT
StSGR1/NYE1	CCAATGAGTGTTATGCCTTGG	TCAACTTTGCTGCTCTTGCAAG
StPP2C31	ACGGATTATGGGACGTGGTA	AGCCAAGGCTAATTTCGTCA
StEIN3/EIL2	CATGATGACATGACGAAGCAAGA	TGCACGATCCTCCAACCTCTACA
StWRKY61	CCAGAGAAACATAAACACAAGAAAC	AGGAGAGACAAAAGAGGGGGAATAA
StLOX3	TTGCTTTACTCCTGGTCGCTACTG	GTTTCAGCCCATGAGGTTGTGTTG

	Transcript ID	Arabidopsis homologs	Tomato homologs	Reference
StNAC030	Soltu.DM.03G029980.1	AT5G07680 *AtNAC4*	Solyc03g115850.2	[1]
StMYB229	Soltu.DM.12G001810.1	AT5G49620 *AtMYB78* AT3G06490 *AtMYB108* AT1G48000 *AtMYB112* AT2G47190 *AtMYB2*	Solyc12g099140.1 *SlMYB45*	[2]
StSGR1/NYE1	Soltu.DM.08G026750.1	AT4G22920 *AtNYE1-SGR1* AT4G11910 *AtNYE2-SGR2*	Solyc08g080090.2 SlSGR1 (NP001234723.1)	[3]
StPP2C31	Soltu.DM.05G023010.3	AT3G11410 *AtPP2CA* AT5G59220 *AtSAG113*	Solyc05g052980.2	[4]
StEIN3/EIL2	Soltu.DM.01G006210.1	AT3G20770 *AtEIN3* AT2G27050 *AtEIL1*	Solyc01g009170.2 *LeEIL2* (AF328785)	[5]
StWRKY61	Soltu.DM.08G028850.1	AT4G23810 AtWRKY53	Solyc08g082110.2 *SlWRKY54*	[6; 7]
StLOX3	Soltu.DM.03G037120.1	AT1G17420 *AtLOX3* AT1G72520 *AtLOX4*	Solyc03g122340.2 *LoxD*	[8]

References:

[1] Singh, A.K., Sharma, V., Pal, A.K., Acharya, V. and Ahuja, P.S. (2013) Genome-wide organization and expression profiling of the NAC transcription factor family in potato (*Solanum tuberosum* L.). DNA Res. 20: 403-23.

[2] Li, X., Guo, C., Ahmad, S., Wang, Q., Yu, J., Liu, C., et al. (2019) Systematic Analysis of MYB Family Genes in Potato and Their Multiple Roles in Development and Stress Responses. Biomolecules 9: 317.

[3] Ma, X., Balazadeh, S. and Mueller-Roeber, B. (2019) Tomato fruit ripening factor NOR controls leaf senescence. J. Exp. Bot. 70: 2727-2740.

[4] Wang, Y., Liao, Y., Wang, Y., Yang, J., Zhang, N., Si, H. (2020) Genome-wide identification and expression analysis of StPP2C gene family in response to multiple stresses in potato (*Solanum tuberosum* L.). J. Integr. Agric. 19: 1609-1624.

[5] Zhang, H., Li, A., Zhang, Z., Huang, Z., Lu, P., Zhang, D., et al. (2016) Ethylene Response Factor TERF1, Regulated by ETHYLENE-INSENSITIVE3-like Factors, Functions in Reactive Oxygen Species (ROS) Scavenging in Tobacco (*Nicotiana tabacum* L.). Sci Rep. 6: 29948.

[6] Huang, Y., Li, M.Y., Wu, P., Xu, Z.S., Que, F., Wang, F., et al. (2016) Members of WRKY Group III transcription factors are important in TYLCV defense signaling pathway in tomato (*Solanum lycopersicum*). BMC Genomics 17: 788.

[7] Zhang, C., Wang, D., Yang, C., Kong, N., Shi, Z., Zhao, P., et al. (2017) Genome-wide identification of the potato WRKY transcription factor family. PLoS One 12: e0181573.

[8] Peivastegan, B., Hadizadeh, I., Nykyri, J., Nielsen, K.L., Somervuo, P., Sipari, N. et al. (2019) Effect of wet storage conditions on potato tuber transcriptome, phytohormones and growth. BMC Plant Biol. 19: 262.
