# Supplementary material for: The protein phosphatase 2A catalytic subunit StPP2Ac2b enhances susceptibility to Phytophthora infestans and senescence in potato
Source: PLoS One. 2022 Oct 10;17(10):e0275844. doi: 10.1371/journal.pone.0275844 (PMC9550054; doi:10.1371/journal.pone.0275844)
Supplement: S2 Table — Transcript IDs of senescence-promoting genes used to construct the heat map of S4 Fig. (PDF) [file pone.0275844.s009.pdf]

	ID (*S. tuberosum* database (http://spuddb.uga.edu/)	Arabidopsis homolog	Functional annotation in *S. tuberosum* database
**1**	Soltu.DM.11G020880.1	AT3G44880 PAO/ACD1 Chlorophyll degradation	Pheophorbide a oxygenase family protein with Rieske [2Fe-2S] domain
**2**	Soltu.DM.12G007710.1	AT4G11910 NYE2 Chlorophyll degradation	non-yellowing
**3**	Soltu.DM.07G010570.1	AT4G13250 NYC1 Chlorophyll degradation	NAD(P)-binding Rossmann-fold superfamily protein
**4**	Soltu.DM.08G026750.1	AT4G22920 AtNYE1 Chlorophyll degradation	non-yellowing
**5**	Soltu.DM.01G026560.2	AT5G13800 PPH Chlorophyll degradation	pheophytinase
**6**	Soltu.DM.02G021370.1	AT5G15250 AtFtsH6 Chlorophyll degradation	FTSH protease
**7**	Soltu.DM.04G037460.1	AT5G42270 FTSH5 Chlorophyll degradation	FtsH extracellular protease family
**8**	Soltu.DM.08G009290.1	AT5G04900 NOL Chlorophyll degradation	NYC1-like
**9**	Soltu.DM.08G022080.2	AT1G32080 AtLrgB Cell death (MP prot)	membrane protein, putative
**10**	Soltu.DM.01G005860.8	AT2G41060 UBA2b Defense	RNA-binding (RRM/RBD/RNP motifs) family protein
**11**	Soltu.DM.04G031660.1	AT1G58340 BCD1 Drug transmembrane transport	MATE efflux family protein
**12**	Soltu.DM.12G025470.1	AT4G29130 HXK1/GIN2 Glucose signal pathway	hexokinase
**13**	Soltu.DM.05G023010.3	AT5G59220 SAG113 Hormone response pathway: ABA	protein phosphatase 2CA
**14**	Soltu.DM.07G013020.1	AT1G19220 ARF19 Hormone response pathway: Auxin	Transcriptional factor B3 family protein / auxin-responsive factor AUX/IAA-related
**15**	Soltu.DM.01G042050.2	AT1G59750 ARF1 Hormone response pathway: Auxin	auxin response factor
**16**	Soltu.DM.10G017720.1	AT1G20330 SMT2/CVP1 Hormone response pathway: BR	sterol methyltransferase
**17**	Soltu.DM.10G020520.1	AT2G38050 DET2/DWF6 Hormone response pathway: BR	3-oxo-5-alpha-steroid 4-dehydrogenase family protein
**18**	Soltu.DM.04G023990.1	AT4G39400 BRI Hormone response pathway: BR	Leucine-rich receptor-like protein kinase family protein
**19**	Soltu.DM.07G022640.1	AT1G04310 ERS2 Hormone response pathway: ET	Signal transduction histidine kinase, hybrid-type, ethylene sensor
**20**	Soltu.DM.11G002410.1	AT1G66330 AAF Hormone response pathway: ET	senescence-associated family protein
**21**	Soltu.DM.01G006210.1	AT3G20770 EIN3 Hormone response pathway: ET	Ethylene insensitive 3 family protein
**22**	Soltu.DM.09G005730.2	AT5G03280 EIN2 Hormone response pathway: ET	NRAMP metal ion transporter family protein
**23**	Soltu.DM.03G037120.1	AT1G72520 LOX4 Hormone response pathway: JA	lipoxygenase
**24**	Soltu.DM.09G024180.1	AT1G55020 LOX1 Hormone response pathway: JA	PLAT/LH2 domain-containing lipoxygenase family protein
**25**	Soltu.DM.09G028490.2	AT2G33150 PED1/KAT2 Hormone response pathway: JA	peroxisomal 3-ketoacyl-CoA thiolase
**26**	Soltu.DM.08G006230.1	AT2G44050 COS1 Hormone response pathway: JA	6,7-dimethyl-8-ribityllumazine synthase / DMRL synthase / lumazine synthase / riboflavin synthase
**27**	Soltu.DM.01G002140.1	AT3G45140 LOX2 Hormone response pathway: JA	lipoxygenase
**28**	Soltu.DM.06G026830.1	AT5G63110 HDA6 Hormone response pathway: JA	histone deacetylase
**29**	Soltu.DM.06G026140.1	AT1G74710 SID2 Hormone response pathway: SA	isochorismate synthase
**30**	Soltu.DM.04G032870.1	AT4G24230 ACPB3 Hormone response pathway: SA	acyl-CoA-binding domain
**31**	Soltu.DM.07G001220.1	AT4G30520 AtSARK Protein degradation/modification	Leucine-rich repeat protein kinase family protein
**32**	Soltu.DM.06G009100.1	AT5G05700 DLS1/ATE1 Protein degradation/modification	arginine-tRNA protein transferase
**33**	Soltu.DM.12G028490.1	AT2G42620 ORE9/MAX2 Protein degradation/modification	RNI-like superfamily protein
**34**	Soltu.DM.03G007830.1	AT1G69270 RPK1 Protein degradation/modification	receptor-like protein kinase
**35**	Soltu.DM.01G049440.1	AT2G21660 ATGRP7 RNA binding	cold, circadian rhythm, and rna binding
**36**	Soltu.DM.06G023430.1	AT1G80350 BOT1 Signal transduction	P-loop containing nucleoside triphosphate hydrolases superfamily protein
**37**	Soltu.DM.12G019640.1	AT2G43790 MPK6 Signal transduction	MAP kinase
**38**	Soltu.DM.06G012820.1	AT2G45660 SOC1 Transcription regulation: MADS	AGAMOUS-like
**79**	Soltu.DM.07G019630.1	AT4G28140 Rap2.4f Transcription regulation: ERF/AP2	Integrase-type DNA-binding superfamily protein
**40**	Soltu.DM.08G028150.1	AT5G41410 BEL1 Transcription regulation: HB	POX (plant homeobox) family protein
**41**	Soltu.DM.07G023850.1	AT3G15030 TCP4 Transcription regulation: TCP	TCP family transcription factor
**42**	Soltu.DM.08G007360.2	AT4G18390 TCP2 Transcription regulation: TCP	TEOSINTE BRANCHED 1, cycloidea and PCF transcription factor
**43**	Soltu.DM.03G034270.1	AT1G49010 AtMYBL Transcription regulation: MYB	Duplicated homeodomain-like superfamily protein
**44**	Soltu.DM.12G001810.1	AT2G47190 ATMYB2 Transcription regulation: MYB	myb domain protein
**45**	Soltu.DM.05G005300.1	AT1G69490 NAP Transcription regulation: NAC	NAC-like, activated by AP3/PI
**46**	Soltu.DM.11G004630.3	AT3G10500 NTL4/NAC053 Transcription regulation: NAC	NAC domain containing protein
**47**	Soltu.DM.03G029980.1	AT3G29035 AtNAC3 Transcription regulation: NAC	NAC domain containing protein
**48**	Soltu.DM.06G025800.1	AT5G39610 ORE1/NAC2 Transcription regulation: NAC	NAC domain containing protein
**49**	Soltu.DM.08G028850.1	AT4G23810 WRKY53 Transcription regulation: WRKY	WRKY family transcription factor
**50**	Soltu.DM.10G025140.1	AT1G13260 RAV1/EDF4 Transcription regulation: AP2/B3	AP2/B3-like transcriptional factor family protein
**51**	Soltu.DM.02G004200.1	AT1G62300 WRKY6 Transcription regulation: WRKY	WRKY family transcription factor
